# Distinct Differentiation Programs Triggered by IL-6 and LPS in Teleost IgM^+^ B Cells in The Absence of Germinal Centers

**DOI:** 10.1038/srep30004

**Published:** 2016-08-02

**Authors:** Beatriz Abós, Tiehui Wang, Rosario Castro, Aitor G. Granja, Esther Leal, Jeffrey Havixbeck, Alfonso Luque, Daniel R. Barreda, Chris J. Secombes, Carolina Tafalla

**Affiliations:** 1 Centro de Investigación en Sanidad Animal (CISA-INIA), Madrid, Spain; 2 Scottish Fish Immunology Research Centre, University of Aberdeen, Aberdeen, UK; 3 Department of Biological Sciences, University of Alberta, Alberta, Canada

## Abstract

Although originally identified as a B cell differentiation factor, it is now known that mammalian interleukin-6 (IL-6) only regulates B cells committed to plasma cells in response to T-dependent (TD) antigens within germinal centers (GCs). Even though adaptive immunity is present in teleost fish, these species lack lymph nodes and GCs. Thus, the aim of the present study was to establish the role of trout IL-6 on B cells, comparing its effects to those induced by bacterial lipopolysaccharide (LPS). We demonstrate that the effects of teleost IL-6 on naïve spleen B cells include proliferation, activation of NF-κB, increased IgM secretion, up-regulation of Blimp1 transcription and decreased MHC-II surface expression that point to trout IL-6 as a differentiation factor for IgM antibody-secreting cells (ASCs). However, LPS induced the secretion of IgM without up-regulating Blimp1, driving the cells towards an intermediate activation state in which antigen presenting mechanisms are elicited together with antibody secretion and expression of pro-inflammatory genes. Our results reveal that, in trout, IL-6 is a differentiation factor for B cells, stimulating IgM responses in the absence of follicular structures, and suggest that it was after follicular structures appeared that this cytokine evolved to modulate TD responses within the GC.

The immune system comprises both innate and adaptive immune responses. While the innate immune system is genetically programmed to detect invariant features of invading microbes, the cells of the adaptive immune system, such as conventional B cells (B2) and T cells, detect specific epitopes through somatically recombined receptors. However, it is now recognized that both branches of immunity are highly interconnected and B cells also possess a certain capacity to directly sense and respond to pathogens though the expression of certain pattern recognition receptors (PRRs) or through the action of cytokines produced by cells of the innate immune system^1^. In general, conventional B cells are activated in response to T-dependent (TD) antigens within the lymphoid follicles and trigger the formation of germinal centers (GCs). These sites promote the close collaboration between proliferating antigen-specific B cells, T follicular helper cells, and the specialized follicular dendritic cells (DCs) that constitutively occupy the central follicular zones of secondary lymphoid organs. In this environment, B cells divide in response to antigens and acquire the capacity to differentiate into antibody-secreting cells (ASCs), reaching a terminal state of plasma cells or memory B cells, both of them with the capacity to secrete high affinity antibodies. This TD pathway provides a strong long-lived immunological memory, but is relative slow to occur. Thus, it must be integrated with additional T-independent (TI) pathways that mainly involve other B cell subsets such as B1 cells or marginal zone (MZ) B cells. These TI responses do not require cooperation from T cells, but instead are much more responsive to products secreted by cells of the innate immune system and have a greater capacity to directly recognize pathogens^1^.

Although evolutionarily jawed fish constitute the first group of animals in which adaptive immunity based on Ig receptors is present^2^, many structural immune peculiarities predict important functional differences between fish and mammalian B cells. The teleost spleen constitutes the main secondary immune organ in the absence of lymph nodes. However, the splenic white pulp is poorly developed in teleosts in comparison to mammals and no GCs are apparent^3^. Regarding mucosal immunity, although fish B cells have been reported in surfaces such as gills, skin, digestive tract and nasal cavities^4^,^5^, they are scattered throughout the mucosa in disorganized lymphoid structures^6^. Additionally, fish contain only three immunoglobulin classes IgM, IgD and IgT (designated as IgZ in some species). IgT is a teleost fish-specific Ig that seems specialized in mucosal immunity^7^,^8^ and IgT^+^ B cells constitute a distinct linage^7^, thus no class switch recombination has ever been reported in fish. As a result, the lack of teleost follicular structures already anticipates that fish B cell responses best resemble mammalian extrafollicular responses. Consequently, teleost B cells share many features of mammalian B1 cells, as for example a high phagocytic capacity^9^,^10^, constitutive expression of many PRRs^4^,^11^ or expression of B1-specific cell markers^12^.

Interleukin 6 (IL-6) is a multi-functional cytokine produced by a wide range of cell types in the early stages of infection. IL-6 modulates a plethora of immune functions through a receptor composed of the restricted IL-6 receptor chain (IL-6R) and a common signal transducer, gp130^13^. Although initially described as a B cell differentiation factor^14^, it was later demonstrated that IL-6 is a potent growth and maturation factor only for cells that have already initiated a differentiation process towards plasma cells, but has minimal capacity to directly induce plasma cell differentiation^15^. Besides, IL-6 enhances antibody production of ASCs but only those that are antigen-specific, whereas non-specific ASCs are unresponsive to IL-6^16^. Interestingly, the normal development of GCs is significantly altered in the absence of IL-6^17^. Consequently, IL-6 deficiency significantly impairs early TD IgG production, but has no effect on IgM TI responses^17^,^18^. Thus, it has been postulated that IL-6 is predominantly involved in the maturation of TD plasma cells in the early stages of GC formation^19^. At a mucosal level, IL-6 also seems implicated in promoting IgA TD responses^20^. Strikingly, the number of B1-derived IgA-secreting cells significantly increases in the absence of IL-6^20^, demonstrating that B1 cells are not regulated by this cytokine and can even be negatively affected by it. Despite this, IL-6 in combination with an anti-IgM antibody induces the expression of surface CD5 on conventional B2 cells that acquire phenotypic characteristics of B1 cells^21^.

Taking into account the main role of IL-6 on TD responses in mammals and the unique attributes of fish B cells, it is important to determine the effect of IL-6 on fish B cells, which we do here using the rainbow trout (*Oncorhynchus mykiss*) as a model. We compare the effects elicited by IL-6 to those triggered by lipopolysaccharide (LPS). LPS, a protein-free endotoxin from the cell wall of Gram-negative bacteria, is the most extensively studied TI antigen in mammals and is directly recognized by B cells through low affinity B cell receptors (BCRs) and PRRs such as Toll like receptors (TLRs)^22^. LPS is not a mitogen for human B cells, since human B cells express neither TLR4 nor CD14, the two canonical ligands for Gram (−) bacterial LPS^23^,^24^. In contrast, mice B cells express TLR4 and are polyclonally activated by it^25^. Interestingly, although TLR4 is thought to be absent from the genome of salmonid fish^26^, some effects of LPS on salmonid B cells have been reported^27^.

Our findings demonstrate that, in contrast to what occurs in mammals, B cells obtained from healthy unstimulated trout respond to IL-6. The effects that IL-6 exerted on these IgM^+^ B cells included proliferation, activation of NF-κB, increased IgM secretion, up-regulation of Blimp1 transcription, increased size and decreased MHC-II surface expression, all pointing to trout IL-6 as a differentiation factor for naïve IgM B cells towards ASCs. Interestingly, LPS also increased IgM secretion but the activation profile was very different to that elicited by IL-6, driving the IgM^+^ B cells towards a pro-inflammatory state. Additionally, IL-6 stimulated B cells mobilized more intracellular calcium in response to BCR cross-linking, demonstrating that IgM ASCs retain a functional BCR on the cell surface. Thus, our results demonstrate that in trout, where follicular structures have not yet been developed, IL-6 regulates IgM B cell responses. This suggests that the emergence of follicular structures marked a critical point in IL-6 evolution, acquiring a novel capacity to specifically regulate TD antibody responses.

## Results

### The IL-6 receptor complex is transcribed in IgM^+^ B cells from naïve fish that are activated by IL-6 stimulation

IL-6 exerts its biological activities through two molecules: the IL-6 receptor, IL-6Rα (also known as gp80 and CD130) and the associated signal transducer glycoprotein gp130. While IL-6Rα is important for ligand binding, gp130 is required for adequate intracellular signaling^28^. Prior to studying the effects of IL-6 on naïve B cells in fish, we determined if these two molecules were constitutively transcribed in sorted naïve IgM^+^ B cells from spleen, blood and kidney, being this last organ the main hematopoietic tissue in fish. We found that IgM^+^ B cells from all three tissues constitutively transcribed IL-6Rα and gp130 at similar expression levels (Supplemental Figure S1A), suggesting that naïve B cells have the capacity to respond to IL-6. These results contrast those of mammals, where only activated B cells express IL-6R^16^,^28^.

To confirm that unstimulated B cells from trout are responsive to IL-6, we first examined the expression of SOCS3 and STAT3, two genes involved in the signaling of IL-6, in sorted IL-6-stimulated trout IgM^+^ B cells. Both SOCS3 and STAT3 were significantly up-regulated in IgM^+^ B cells in response to IL-6 stimulation (Supplemental Figure S1B), as previously reported in other IL-6-responsive cell types in trout^29^. LPS, on the other hand, induced the transcription of STAT3 but had no effect on SOCS3 mRNA levels (Supplemental Figure S1B). To further confirm these results and demonstrate a direct effect of IL-6 on IgM^+^ B cells, we sorted IgM^+^ cells and afterwards incubated them with IL-6 or LPS. After 1 h of incubation, the phosphorylation of STAT3 was confirmed through Western blot in response to IL-6 and LPS (Supplemental Figure S1C). These results confirm a direct effect of IL-6 on IgM^+^ B cells.

### IL-6 induces IgM^+^ cell proliferation

**Figure 1:**
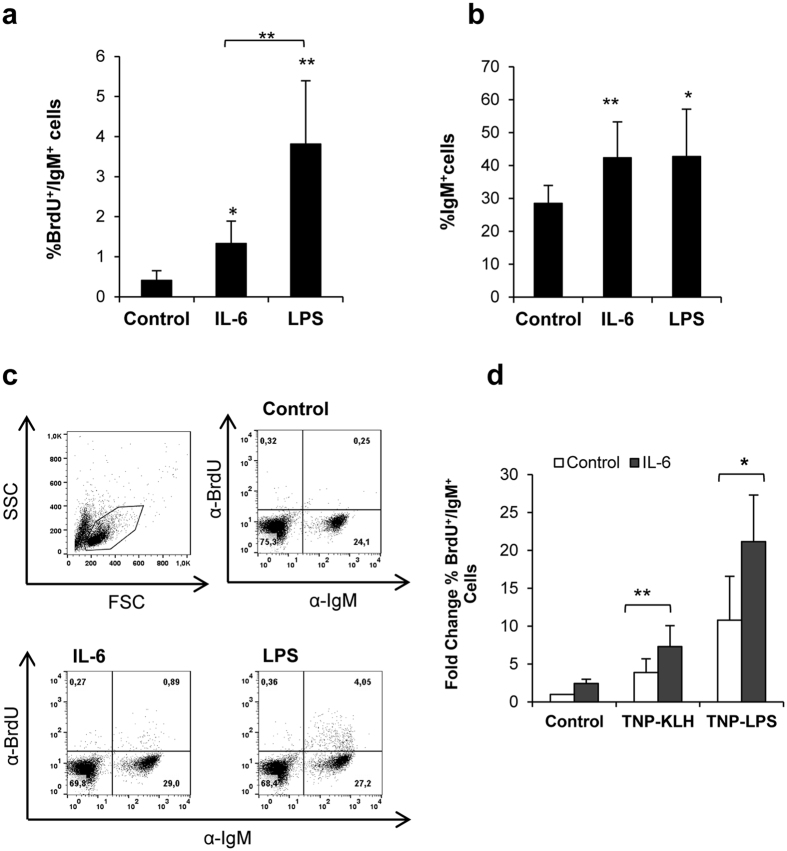
IL-6 and LPS elicit B cell proliferation and increased survival. Spleen leukocytes were incubated with IL-6 (200 ng/ml) or LPS (100 μg/ml) for 3 days at 20 °C. After this time, BrdU was added to the cultures and incubated for a further 24 h. The percentage of proliferating (BrdU^+^) IgM^+^ B cells was determined as described in Material and Methods. (**a**) Percentage (mean + standard deviation) of proliferating IgM^+^ B cells (BrdU^+^/IgM^+^) after treatment with IL-6 and/or LPS (n = 6). (**b**) IgM^+^ B cell survival estimated as percentage of IgM^+^ cells (proliferating and non-proliferating cells) in cultures (mean + standard deviation) (n = 6). (**c**) Representative dot plots are shown. (**d**) Spleen leukocytes were stimulated with TNP-KLH (5 μg/ml) or TNP-LPS (5 μg/ml) in the presence or absence of IL-6 (200 ng/ml). The percentage of proliferating IgM^+^ cells was assessed as described above. Data are shown as the mean fold change relative to the control value for unstimulated controls + standard deviation (n = 5). Asterisks denote significant differences between cells treated with IL-6 or LPS and their corresponding controls and between IL-6 and LPS treated cells when indicated. *P < 0.05, **P < 0.01.

As already mentioned, mammalian IL-6 has no effect on naïve B cells, but has been shown to induce the proliferation of pre-B cells^30^ as well as plasmablasts^31^. In contrast, LPS is a potent inducer of B cell proliferation in mice and fish^27^,^32^. Thus, we compared the lymphoproliferative effects of LPS to those of IL-6 in trout splenocytes. Our results show that trout spleen IgM^+^ B cells significantly proliferated in response to IL-6 (Fig. 1a,c), although at levels significantly lower than those elicited by LPS (Fig. 1a,c). When the percentage of IgM^+^ B cells was evaluated in these cultures without taking into account BrdU uptake, the percentage of IgM^+^ cells was quite similar in LPS- and IL-6-treated cultures, suggesting an additional positive effect of IL-6 on B cell survival (Fig. 1b). The proliferative and survival effects observed were specific to IL-6 since another cytokine produced and tested in parallel, IL-4/13A, was unable to increase the number of trout IgM^+^ cells during the *in vitro* culture^33^.

Since in mammals IL-6 only activates B cells previously stimulated with TD antigens^17^,^18^, we also evaluated the potential synergistic effect of IL-6 on IgM^+^ B cell proliferation induced by a TD antigen (TNP-KLH) or a TI antigen (TNP-LPS). We performed this experiment because despite their lack of follicular structures, fish can orchestrate responses to TD antigens^34^, possibly through a mechanism that resembles rare extrafollicular TD responses reported in mammals^35^,^36^,^37^. For this, we incubated splenocytes with TNP-KLH or TNP-LPS in the presence or absence of IL-6 and evaluated the percentage of BrdU^+^/IgM^+^ B cells after 4 days. We observed that the addition of IL-6 could further enhance the proliferative effects provoked by TNP-LPS or TNP-KLH (Fig. 1d), indicating that, in trout, IL-6 can have regulatory effects on both TD and TI responses.

### IL-6 up-regulates IgM secretion in naïve B cells

**Figure 2:**
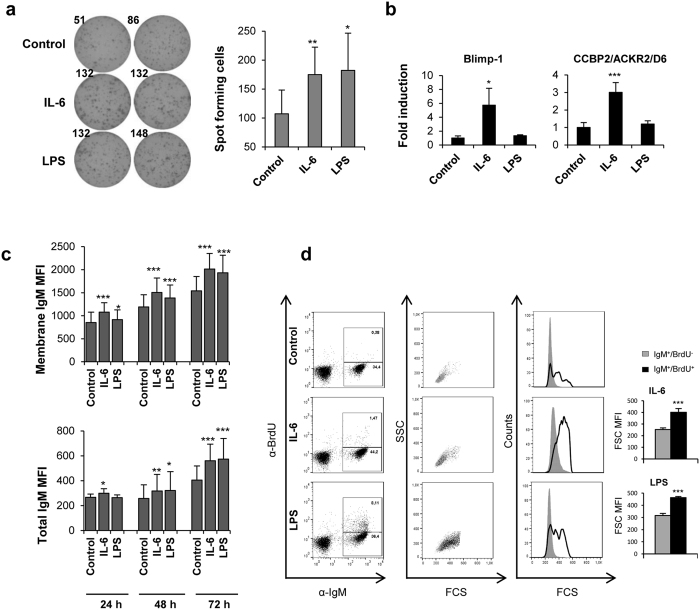
IL-6 and LPS activate IgM secretion in naïve B cells. (**a**) ELISPOT analysis of IgM-secreting cells in splenocyte cultures treated with IL-6 (200 ng/ml), LPS (100 μg/ml) or non-stimulated. Splenocytes were cultured for 3 days in ELISPOT plates previously coated with anti-trout IgM mAb (2 μg/ml) in the presence or absence of the different stimuli. After incubation, cells were washed away and a biotinylated anti-trout IgM mAb (1 μg/ml) was used to detect numbers of spot forming cells. Duplicates from a representative experiment (left) and quantification of spot forming cells (right) from 5 independent experiments are shown (mean + standard deviation). (**b**) Spleen leukocytes were incubated with media containing IL-6, LPS or control media alone for 24 h at 20 °C. After that time, IgM^+^ B cells were sorted using an anti-trout IgM mAb and RNA was extracted. Relative transcript expression of Blimp-1 and ACKR2 is shown (mean + standard deviation, n = 6). (**c**) Membrane IgM and total IgM expression of IgM^+^ cells after incubation with IL-6, LPS or control media for 24, 48 and 72 h. Mean fluorescence intensity (MFI) + standard deviation is shown (n = 9). (**d**) Dot plots and histograms showing the Forward scatter (FSC) from IgM^+^ B cells and BrdU^+^/IgM^+^ B cells incubated in the presence or absence of LPS or IL-6, from one representative experiment. Graphs showing FSC MFI values from 8 independent experiments (mean + standard deviation) are included next to the histograms for stimulated cultures. *P < 0.05, **P < 0.01, ***P < 0.001.

In mammals, IL-6 is unable to induce differentiation of naïve B cells towards plasmablasts on its own^15^. In contrast, the *in vitro* incubation of naïve splenic B cells with IL-6 was sufficient to induce the secretion of IgM, as verified in an ELISPOT assay (Fig. 2a,b). When this experiment was carried out in previously immunized fish, IL-6 also increased the secretion of IgM, at levels significantly higher than those observed in non-immunized healthy trout (Supplemental Figure S2). As previously reported^27^, LPS also induced the secretion of IgM by trout B cells, however, our results suggest that the mechanisms through which IgM secretion is induced differ between IL-6 and LPS-stimulated B cells. At the transcript level, only IL-6 up-regulated the transcription of Blimp1, a protein required for the development of ASCs and the maintenance of long-lived plasma cells^38^, while LPS had no effect (Fig. 2b). Interestingly, IL-6 also up-regulated the transcription of ACKR2, an atypical chemokine receptor specific for innate B cells^39^, while LPS showed no effect (Fig. 2b). Thus, our results demonstrate that, unlike mammalian IL-6, fish IL-6 is a differentiation factor for naïve B cells towards an ASC profile. On the other hand, LPS seems to induce the secretion of IgM in a Blimp1-independent fashion.

In mammals, the differentiation of B cells to plasma cells provokes a down-regulation of membrane Igs that completely loose the BCR when terminally differentiated^40^. However, recent reports have demonstrated that this seems to only be true for IgG plasma cells, since fully differentiated IgM or IgA-secreting plasma cells retain a functional BCR in the cell membrane^41^. In our studies, we observed that both IL-6 and LPS increased the levels of expression of total IgM in B cells (Fig. 2c), along with increased membrane IgM levels (Fig. 2c). Therefore, these IL-6 or LPS-induced plasmablasts retain and even up-regulate IgM levels in the cell surface while increasing their secretion of IgM, similar to mammalian IgM plasma cells. The secretion of IgM in response to LPS or IL-6 was associated with an increase in the size of IgM^+^ cells, as shown by increased forward side scatter (FSC) in stimulated cells in comparison to control cells. This increase in size was significant when proliferating cells were analyzed (Fig. 2d) demonstrating that LPS and IL-6 induce the proliferation of IgM^+^ B cells and part of the progeny differentiate into ASCs that retain IgM on the cell surface.

### IL-6 activates NF-κB in spleen IgM^+^ B cells

**Figure 3:**
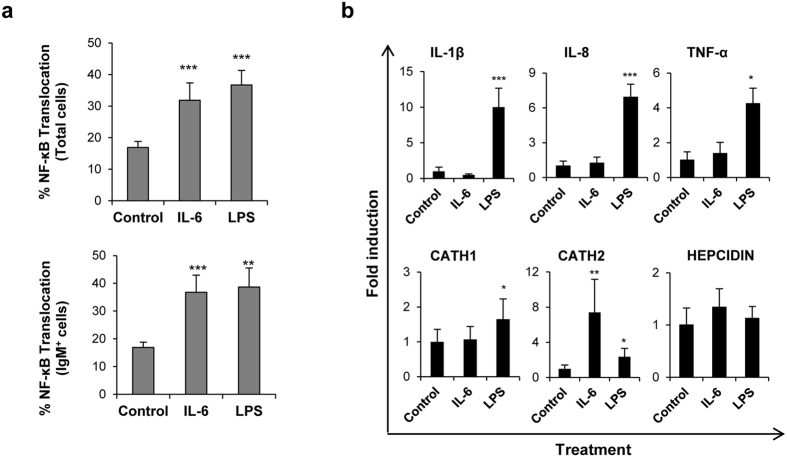
Effect of IL-6 and LPS on NF-κB activation and transcription of pro-inflammatory and antimicrobial genes in B cells. (**a**) Translocation of NF-κB to the nucleus in IgM^+^ splenocytes following stimulation with LPS (100 μg/ml) or IL-6 (200 ng/ml) for 24 h. The mean percentages of NF-κB translocation in total cells (upper panel) and IgM^+^ B cells (lower panel) from six independent experiments are shown. **P < 0.01, ***P < 0.001. (**b**) Spleen leukocytes were incubated with IL-6, LPS or control media alone for 24 h at 20 °C. The effect of IL-6 and LPS on the transcription of IL-1β1, IL-8, TNF-α3, cathelicidin-1 (CATH1), CATH2 and hepcidin was then studied in sorted IgM^+^ B cells from these cultures. The relative transcript expression (mean + standard deviation) of 6 independent experiments is shown. *P < 0.05, **P < 0.01, ***P < 0.001.

After binding to its receptor, IL-6 activates gene expression mainly through the STAT3 pathway^28^,^42^ and, in line with its pro-inflammatory nature, it has been shown to activate NF-κB in intestinal epithelial cells^43^. Despite this, whether IL-6 activates NF-κB in mammalian B cells has not been established. In mice, LPS induces a dual BCR/TLR-signaling by engaging TLR4 through the lipid A moiety and the BCR through the polysaccharidic moiety^44^. As a result, both the canonical and non-canonical NF-κB signaling pathways are activated in B cells in response to LPS. To determine whether IL-6 and LPS activate the NF-κB signaling pathway in our system, we analyzed the translocation of the NF-κB p65 subunit to the nucleus of IgM^+^ B cells using a quantitative imaging flow cytometry-based approach which we have used previously^11^. We observed a significant increase in the percentage of total splenocytes and IgM^+^ B cells in which p65 translocated to the nucleus in response to IL-6 and LPS in comparison with control cells, indicating that both stimuli activate the canonical NF-κB pathway (Fig. 3a).

However, when the transcriptional activity of NF-κB-dependent genes such as IL-1β1, IL-8 or TNF-α3 was studied, only LPS was capable of inducing a significant up-regulation of their mRNA levels (Fig. 3b). Similarly, differential effects of LPS and IL-6 in the transcriptional activation of antimicrobial peptides was also observed. While LPS up-regulated cathelicidin 1 (CATH1) mRNA levels (Fig. 3b) CATH2 was significantly up-regulated by both LPS and IL-6, although the effect of IL-6 was much stronger (Fig. 3b). Taken together, these results show that although IL-6 and LPS activate NF-κB in IgM^+^ B cells, the downstream effects of this activation are different.

### IL-6 and LPS-stimulated IgM^+^ B cells are predisposed for BCR-mediated calcium mobilization

**Figure 4:**
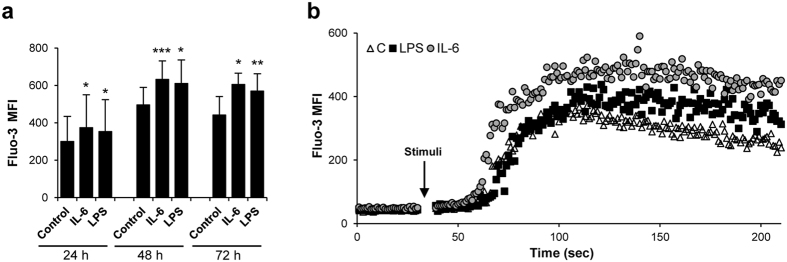
IL-6 and LPS predispose IgM^+^ B cells to calcium mobilization after BCR cross-liking. Spleen leukocytes were incubated with IL-6 (200 ng/ml), LPS (100 μg/ml) or control media alone for 24, 48 and 72 h at 20 °C. After the different incubation periods, cells were loaded with Fluo-3 AM (5 μM final concentration), and its baseline emission was measured by flow cytometry for 30 s, and then stimulated with 0.5 ug/ml of Alexa 647 conjugated anti-IgM mAb. Fluorescence was then measured for a further 180 s. (**a**) Mean fluorescence intensity (MFI) plus standard deviation of intracellular Ca^2+^ levels (Fluo-3) in IgM^+^ B cells is shown (n = 6). *P < 0.05, **P < 0.01, ***P < 0.001. (**b**) Scatter plot showing Fluo-3 MFI levels in spleen IgM^+^ B cells after 48 h of incubation with the appropriate stimuli.

Anti-IgM stimulation of B cells mimics the recognition of a high affinity antigen by the BCR, consequently leading to a rapid increase of intracellular calcium^45^. This calcium mobilization plays an important role in B cell activation and is required for correct downstream signaling of the BCR^45^. Thus, we also studied the effect of anti-IgM cross-linking of the BCR in splenocyte cultures that had been previously stimulated with IL-6 or LPS and compared them to those in non-stimulated control cells, given the fact that IL-6 or LPS-stimulated cells seem to retain their BCR. As expected, anti-IgM induced a rapid mobilization of intracellular calcium, and furthermore, the levels of calcium released upon anti-IgM stimulation were significantly higher in cells that had been previously stimulated with IL-6 or LPS (Fig. 4a,b). This stimulatory effect was observed after 24, 48 or 72 h of stimulation. Thus, our results reveal that IL-6 renders the cells more responsive to BCR engagement.

### IL-6 down-regulates the surface expression of MHC-II in naïve trout B cells

**Figure 5:**
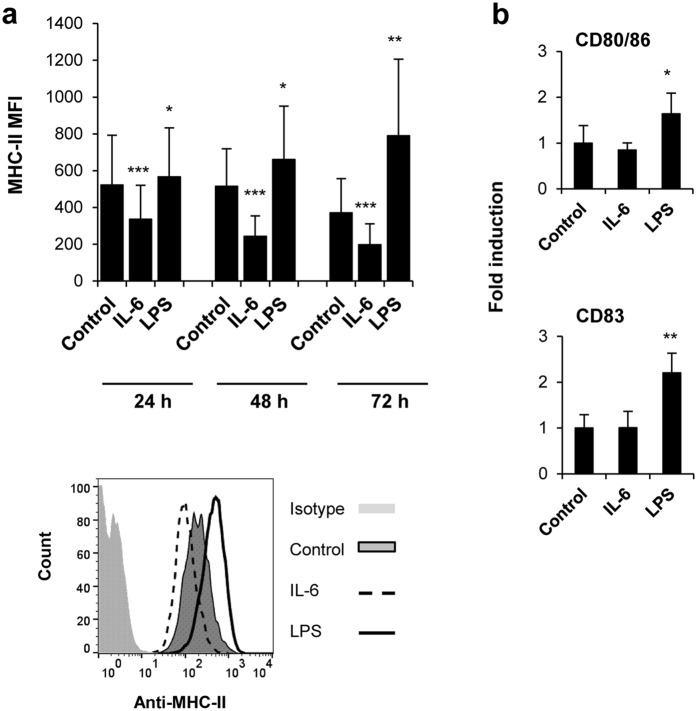
Differential effects of IL-6 and LPS on antigen presenting properties of IgM^+^ B cells. Spleen leukocytes were incubated with IL-6 (200 ng/ml), LPS (100 μg/ml) or control media alone for 24, 48 and 72 h at 20 °C. The levels of MHC-II expression on the surface of IgM^+^ B cells were then measured via flow cytometry using a specific mAb against trout MHC-II. (**a**) MFI + standard deviation from 6 independent experiments. *P < 0.05, **P < 0.01, ***P < 0.001. Representative histogram from one experiment shown below. (**b**) Spleen leukocytes were incubated with IL-6, LPS or control media alone for 24 h at 20 °C. The effect of IL-6 and LPS on the transcription of CD80/86 and CD83 co-stimulatory molecules was then studied in sorted IgM^+^ cells from these cultures. The relative transcript expression (mean + standard deviation) of 5 independent experiments is shown. *P < 0.05, **P < 0.01.

The response of B cells against an antigen not only requires antigen binding to and signaling through the BCR but also the processing and presentation of the BCR-bound antigen to helper T cells in the context of MHC-II^46^. Thus, we also determined the effect of IL-6 or LPS stimulation on the levels of expression of surface MHC-II of naïve B cells using a specific anti-trout MHC-II antibody. We observed that IL-6 provoked a significant decrease of surface MHC-II expression that was detectable from 24 h post-stimulation up to 72 h post-stimulation (Fig. 5a). In contrast, LPS-stimulated B cells showed increased surface MHC-II levels at all time points studied (Fig. 5a). Along with this increase in MHC II levels, LPS also up-regulated the levels of transcription of two co-stimulatory molecules: CD80/86, a molecule with similar homologies to mammalian CD80 and CD86^47^, and CD83 (Fig. 5b). IL-6, on the other hand, had no effect on their transcription levels (Fig. 5b).

### IL-6 has no effect on the phagocytic activity of IgM^+^ B cells

**Figure 6:**
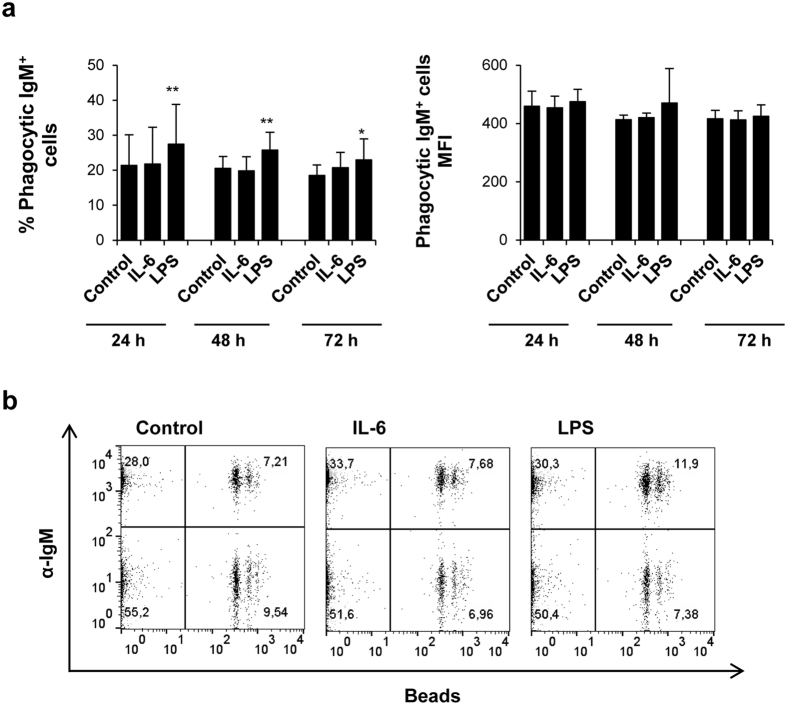
IL-6 has no effect on the phagocytic capacity of IgM^+^ B cells. Splenocytes were cultured in the presence of IL-6 (200 ng/ml) or LPS (100 μg/ml) for 24, 48 or 72 h at 20 °C. Non-stimulated controls were also included. After the different incubation periods, cells were exposed to fluorescent beads for a further 3 h at 20  °C. Non-ingested beads were removed by centrifugation over a cushion of 3% (w/v) BSA in PBS supplemented with 4.5 (w/v) D-glucose (Sigma). (**a**) Data are shown as mean percentage of phagocytic IgM^+^ B cells (left) or Mean fluorescence intensity (MFI) (right) + standard deviation from six independent fish. *P < 0.05, **P < 0.01. (**b**) Representative dot plots for each condition.

Since trout B cells have a potent phagocytic activity^9^, we also investigated whether IL-6 or LPS pre-stimulation of naïve IgM^+^ B cells could have an effect on their phagocytic activity. For this, splenocytes were incubated with IL-6 or LPS for different incubation periods, or left-unstimulated in the same conditions. Thereafter, 1 μm polystyrene-based fluorescent beads were added to the cultures and after 3 h of incubation the phagocytic activity of IgM^+^ cells was determined in flow cytometry. We observed that while pre-stimulation of B cells with LPS significantly increased the percentage of phagocytic IgM^+^ B cells in spleen, pre-stimulation with IL-6 produced no effect (Fig. 6a,b).

### IL-6 has a synergistic effect on antigen-specific IgM responses *in vivo*

**Figure 7:**
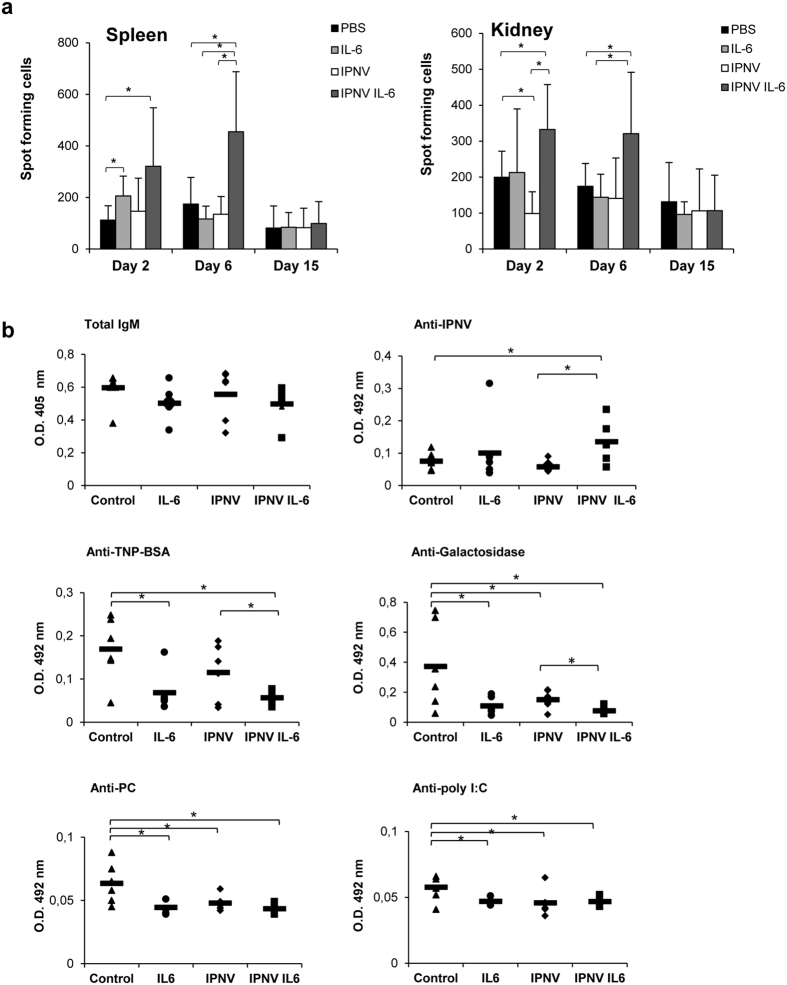
Synergistic effects of IL-6 on antigen-induced *in vivo* IgM production. Rainbow trout were injected i.p. with 100 μl of PBS, 100 μl of PBS with IL-6 (100 ng/fish), 100 μl of PBS with 2 × 10^10^ TCID_50_/ml inactivated IPNV, or 100 μl of PBS containing the same amount of IPNV and IL-6. (**a**) The number of IgM secreting cells was evaluated at days 2, 6 and 15 post-injection in spleen and kidney leukocyte cultures by ELISPOT as described in the Materials and methods section (mean + standard deviation; n = 6). Asterisks indicate significant differences between groups as indicated. *P < 0.05. (**b**) Total IgM, IPNV-specific and natural (BSA; galactosidase; PC, phosphorylcholine; and poly I:C-specific) IgM titers were measured in the sera of fish killed at day 15 post-immunization by ELISA. Results are shown as absorbance at 405 nm (for total IgM) or absorbance at 492 nm (for specific IgMs) for individual fish. Bars indicate mean values in each group and asterisks denote significant differences between groups as indicated. *P < 0.05.

Given the stimulatory effects observed for IL-6 on IgM^+^ B cells *in vitro*, we next investigated whether this stimulatory effect on naïve IgM^+^ B cells could also condition IgM responses elicited *in vivo* in response to a specific antigen. For this, we immunized fish with inactivated infectious pancreatic necrosis virus (IPNV) either alone or in combination with IL-6. Control groups treated with IL-6 alone or PBS were also included. The administration of IL-6 alone significantly increased the number of IgM-secreting cells in the spleen at day 2, but no effect was observed in the following days (Fig. 7a). IPNV on its own was unable to increase the number of IgM-secreting cells in spleen or kidney when compared to controls, however when IPNV was combined with IL-6, the number of IgM-secreting cells significantly increased in spleen at day 6 post-immunization and in the kidney at day 2 post-immunization, when compared to controls or fish injected with IPNV alone (Fig. 7a). These positive effects of IL-6 on the number of ASCs elicited by IPNV, correlated with significantly increased IPNV-specific IgM titers in sera of fish injected with IPNV and IL-6 when compared to titers in fish injected with IPNV alone (Fig. 7b). Surprisingly, this increase in IPNV-specific IgM had no effect on total IgM titers (Fig. 7b) at the time point sampled (day 15 post-immunization).

To get additional information on the antibodies present in the sera we also analyzed the titers of natural antibodies against four classic antigens, BSA, galactosidase, phosphorylcholine and dsRNA (poly I:C) in the same groups. We observed a significant decrease in the titers of antibodies that bind these four antigens in all the groups treated with IL-6 or/and IPNV. Interestingly, at the same time that IL-6 significantly increased IPNV-specific IgM titers when compared to those elicited by IPNV alone (Fig. 7b), it decreased the amount of natural antibodies reactive against BSA or galactosidase in serum (Fig. 7b). Taken together, our results strongly suggest that when combined with an antigen *in vivo*, IL-6 preferentially activates antigen-specific B cells as occurs in mammals.

## Discussion

Although mammalian IL-6 exclusively signals in antigen-experienced cells^15^, thus only affecting the outcome of switched antibody isotypes such as IgG or IgA^18^,^19^,^48^; a recent study reported that fugu (*Takifugu rubripes*) unstimulated B cells transcribe both IL-6Rα and gp130 and that the *in vitro* stimulation of blood leukocytes with IL-6 up-regulates IgM transcription levels^42^. These results suggest that fish B cells from healthy unstimulated fish could be responsive to IL-6. Hence, we decided to investigate further the capacity of IL-6 to regulate B cell activity in fish, using the rainbow trout as a model. Once we had verified that rainbow trout unstimulated IgM^+^ B cells also transcribe IL-6Rα and gp130, we examined the effects of IL-6 on different relevant functions of fish B cells. We compared the effects provoked by IL-6 to those elicited by LPS, since there is still some controversy about the degree to which trout cells respond to LPS. Although LPS stimulation was reported to increase IgM secretion and lymphocyte proliferation in trout^27^, TLR4 seems absent from salmonid genomes^26^. In this context, our studies would help to establish how, in the absence of follicular structures, fish IL-6 regulates the activity of IgM^+^ B cells. Furthermore, our results would provide additional evidence as to how teleost B cells respond to LPS.

LPS is a potent mitogen of B cells and the *in vitro* responses of these cells to LPS are very robust both in mice^32^ and in fish^49^. In our experiments, LPS and IL-6 were both mitogenic for trout IgM^+^ cells. Although the proliferation levels in response to IL-6 were significantly lower than those observed for LPS, the percentage of viable IgM^+^ B cells in the cultures was similar in response to the different stimuli and significantly higher than the percentage observed in control cultures, suggesting that non-proliferating B cells have an increased survival in IL-6-treated cultures, even higher than that observed in LPS-treated cultures. In mammals, while some authors have reported a proliferative effect of IL-6 on pre-activated B cells^50^, other researchers have described stimulation of Ig secretion in the absence of proliferative responses^51^,^52^. However, even in those cases where proliferating effects have been reported, they seem to be associated only with previously activated cells and are never seen in unstimulated B cells. Although it could be possible that some of the B cells in our cultures have been previously exposed to an antigen, it seems unlikely that all the B cells responding in our experiments are pre-activated cells given the fact that these cells express IgD on the cell membrane and transcribe surface IgM and IgD at high levels (data not shown). On the other hand, even though we stimulated B cells in cultures in which other cells were present during the incubation period, we have demonstrated that STAT3 is phosphorylated in IgM^+^ B cells incubated with IL-6 in the absence of other cell types. Likewise, it should be noted that there was no significant proliferation of IgM^−^ cells in these cultures (Fig. 1c) and that even if IgM^+^ B cells already accounted for approximately 30% of the leukocyte population in the spleen, this percentage was increased to approximately 50% in IL-6-treated cultures after 4 days. All the evidence points to a preferential effect of IL-6 on a quite abundant population of B cells within the spleen of unstimulated fish. In addition to these experiments in which leukocytes were treated with IL-6 alone, we also determined the effect of IL-6 on the B cell proliferation induced by antigen encounter, and again observed stimulatory effects. These synergistic effects were visualized in response to either TD (TNP-KLH) or TI (TNP-LPS) antigens, unlike the situation in mammals where IL-6 only seems to promote TD responses^17^,^18^. Thus, trout IL-6 has positive effects on proliferation and survival of B cells stimulated with TD antigens, and in contrast to mammalian IL-6, it also has mitogenic effects on trout naïve B cells and cells activated with TI antigens.

IL-6 was originally reported as a cytokine capable of inducing antibody production in B cell lines, augmenting the secretion of both IgM and IgG on Epstein-Barr virus transformed B cells^51^,^52^. However, later reports demonstrated a preferential effect of IL-6 on switched antibody isotypes, given the fact that IL-6 only plays a role in the terminal differentiation of antigen-experienced B cells^15^. Thus, IL-6 over-expression in mice provokes IgG plasmacytosis, but has no effects on plasma cells of other Ig isotypes^53^,^54^. Likewise, IL-6-deficient mice showed reduced antigen-specific IgG1, IgG2a and IgG3 levels after immunization with a TD antigen, but IgM levels were unaffected^17^. IL-6 also supports the differentiation of IgA-secreting plasma cells in different mucosal surfaces^55^,^56^. Teleost fish do not express IgG, IgA or IgE and rely on non-switched IgM, IgD and IgT responses to fight infections. In this context, trout IL-6 was capable of inducing IgM secretion in spleen B cells, at a level comparable to that induced by LPS. This increased IgM secretion in response to IL-6 was evidenced in ELISPOT and flow cytometry and the fact that these IL-6 stimulated cells increased in size and up-regulated Blimp1 further supports our observations indicating that trout IL-6 has the capacity to induce the differentiation of unstimulated B cells to IgM ASCs. Interestingly, LPS also provoked a size increase and augmented IgM secretion but without induction of Blimp1, suggesting that either LPS exerts its activity on a different B cell population to IL-6 or alternatively it acts on the same population but drives them towards a different activation state. In mammals, B2 cells differentiate into ASCs in response to LPS along with Blimp1 up-regulation, however, there is some controversy as to whether B1 cells require Blimp1. While some studies revealed that mammalian B1 cells secrete IgM independently of Blimp1^57^, the IgM production by B1 cells in Blimp1-deficient animals was inhibited, suggesting the requirement of Blimp1 for normal IgM production^58^.

To further study the effects of IL-6 on IgM secretion, we performed additional experiments *in vivo*. Here again, the *in vivo* administration of IL-6 alone significantly increased the number of IgM-secreting cells in the spleen at day 2 post-injection. This effect was not observed in the kidney and was lost in the spleen at later sampling points, revealing a tissue-specific transitional effect. In mammals, some of the positive effects that IL-6 has on IgG expression are also transitional, since IL-6 is required for the normal induction, but not for the maintenance of plasma cell responses *in vivo* because the effects of different survival factors are redundant^59^. Additionally, we studied how IL-6 affected the antibody response elicited by the injection of an IPNV vaccine, observing that although IPNV by itself was unable to induce a significant IgM response, when combined with IL-6, the number of IgM-secreting cells in the spleen or head kidney significantly increased in comparison to fish immunized with IPNV alone. Although the stimulatory effect on the number of IgM-secreting cells was no longer visible at day 15 post-injection, at this point we could still see a synergistic effect of IL-6 on the amount of IPNV-specific antibodies in serum. While total IgM levels were not significantly higher in fish injected with IPNV and IL-6, the increase in the amount of IPNV-specific IgM went along with a significant decrease in the quantity of IgM with alternative specificities, such as BSA- or galactosidase-specific IgM. Increases in antigen-specific IgG titers with constant total IgG titers have also been reported in humans and the authors postulated that specific IgG levels were increased without having an effect on total IgG titers because the ratio of antigen-specific cells was very low in relation to the total pool of B cells^60^. Similarly, it seems that trout IL-6, when combined with IPNV, increases IgM secretion of a discrete number of B cells with no effect on overall IgM production. Furthermore, it seems that the negative effect of IL-6 on the production of natural antibodies is a conserved effect, as antibodies against phosphorylcholine and LPS are increased in IL-6 gene knockout mice^20^. Taken together, our results clearly demonstrate that IL-6 is able to enhance antigen-specific IgM responses in fish as observed in mammals for switched Ig isotypes^17^,^53^,^55^,^56^.

When mammalian B cells start their differentiation towards plasma cells, a secretory switch in the mRNA provokes the down-regulation of surface Ig and an increase of secreted Ig, resulting in the lack of BCR once the cells are fully differentiated^40^. However, recent studies have demonstrated that whilst this phenomenon occurs in IgG plasma cells it is not seen in IgM or IgA-secreting plasma cells, as the latter preserve a functional BCR in the cell surface even if fully differentiated^41^. Similarly, we observed that the IL-6- or LPS-induced differentiation of trout B cells to IgM ASCs was not associated with a decrease in surface IgM levels. As hypothesized in mammals^41^, the presence of a functional BCR on IgM ASCs would allow them to respond directly to their specific antigen upon secondary encounters, unlike IgG ASCs. The fact that IL-6 and LPS-activated B cells retain a functional BCR, despite their differentiation to ASCs was confirmed by the mobilization of intracellular calcium in response to anti-IgM cross-linking. Interestingly, these stimulated cells mobilized intracellular calcium at levels significantly higher than control B cells, demonstrating that IL-6 and LPS predispose B cells to a posterior signaling through the BCR despite their differentiation to ASCs. This is the first time such an effect is reported for IL-6 in B cells and consequently it would be interesting to determine the effect of mammalian IL-6 on BCR signaling in IgM- and IgA-secreting plasma cells.

The transcription factor NF-κB is critically involved in many cellular processes such as inflammation, immune response, proliferation or apoptosis^61^. In our experiments, we have established that IL-6 and LPS canonically activate NF-κB in IgM^+^ B cells from unstimulated fish. In mammals, BCR cross-linking, CD40 ligation^62^ or stimulation with LPS or phorbol ester^63^ have been shown to activate NF-κB in B cells. However, to our knowledge, this is the first report of IL-6-mediated NF-κB activation in B cells, although IL-6 has been shown to activate this transcription factor in epithelial cells^43^. As a consequence of NF-κB activation, different immune genes should be transcriptionally up-regulated. However, only LPS induced the transcription of typically NF-κB-regulated genes such as IL-1β, IL-8 and TNF-α, suggesting that although IL-6 activated cells translocate p65 to the nucleus, additional cellular mechanisms modify the downstream effects. Of course, the up-regulation of IgM synthesis observed in IL-6-stimulated B cells could be regulated by NF-κB as the promoter of the IgM light chain is one of the main targets for NF-κB in mammals^64^. Since it is known that different NF-κB components can be activated through a variety of mechanisms with different downstream effects^61^, it seems possible that IL-6 and LPS are modulating distinct NF-κB activation pathways, and this should be studied further.

As trout IL-6 induced the transcription of several antimicrobial peptides in trout macrophages^29^ and taking into account that B cells in fish have antimicrobial properties associated to their phagocytic activity^9^, we also studied the effect of IL-6 and LPS on CATH-1, CATH-2 and hepcidin transcription. While CATH-1 and CATH-2 were up-regulated by LPS, IL-6 only up-regulated CATH-2, as occurred in macrophages^29^. Despite the fact that IL-6 modulates hepcidin transcription in macrophages^29^, IL-6 had no effects on hepcidin expression in IgM^+^ B cells. Nevertheless, these results highlight the potent antimicrobial properties of fish B cells. Similar to other antigen presenting cells, B cells express MHC II on the cell surface. Thus, we determined the effects of IL-6 and LPS on MHC II surface expression levels and the phagocytic capacity of trout B cells. Again the effects exerted by IL-6 and LPS were quite different, all of them summarized in Table 1. While IL-6 significantly down-regulated MHC-II surface expression on B cells, as expected in an ASC^65^, LPS provoked a significant up-regulation of MHC-II expression and increased mRNA levels of the co-stimulatory molecules CD80/86 and CD83. The activation of IL-6/STAT3 also induces the suppression of antigen presentation in human dendritic cells^66^. In contrast, and as seen in our studies, mammalian B cells stimulated with LPS up-regulate MHC-II surface expression together with co-stimulatory molecules (CD86, CD40)^67^ and plasma cells differentiated in response to TI antigens retain MHC-II expression and a functional antigen presenting machinery^68^. Thus, it seems that plasma cells generated in response to LPS or other TI antigens, are not exclusively specialized in antibody secretion and still play a role in other immune functions such as antigen presentation and pathogen clearance, and in line with this hypothesis, LPS provoked a slight but significant increase in the number of phagocytic B cells in the spleen cultures. This increase was observed from 24 to 72 h post-treatment, whereas IL-6 had no effect. Since mammalian B1 cells are also phagocytic, it would be interesting to determine if LPS is capable of increasing their phagocytic capacity.

Overall our results demonstrate that in contrast to mammals, fish IL-6 can trigger unstimulated B cells to initiate their differentiation towards ASCs. Consequently, trout IL-6 has mitogenic effects on IgM^+^ cells and these proliferating cells increase in size, augment their secretion of IgM, up-regulate Blimp1 and decrease their surface MHC-II expression. On the other hand, LPS shows a potent mitogenic activity and increases the secretion of IgM, while driving the cells towards a quite different profile that predicts additional functions such as increased antigen presentation (given the increased MHC-II surface expression, up-regulated transcription of co-stimulatory molecules and higher phagocytic activity) and a pro-inflammatory role (through the up-regulation of IL-1ß, IL-8 and TNF-α). These results highlight that IgM secretion can be induced through quite distinct differentiation pathways in fish.

## Methods

### Animals

Healthy specimens of female rainbow trout (*Oncorhynchus mykiss*) of approximately 50–70 g were obtained from Centro de Acuicultura El Molino (Madrid, Spain). Fish were maintained at the Animal Health Research Center (CISA-INIA) laboratory at 14 °C with a re-circulating water system and 12:12 h light:dark photoperiod. Fish were fed twice a day with a commercial diet (Skretting, Spain). Prior to any experimental procedure, fish were acclimatized to laboratory conditions for 2 weeks and during this period no clinical signs were ever observed. The experiments described comply with the Guidelines of the European Union Council (2010/63/EU) for the use of laboratory animals and were previously approved by the Ethics committee from the Instituto Nacional de Investigación y Tecnología Agraria y Alimentaria (INIA; Code CEEA 2011/044).

### Leukocyte isolation

Rainbow trout were killed via benzocaine (Sigma) overdose and blood was extracted with a heparinized needle from the caudal vein and diluted 10 times with Leibovitz medium (L-15, Life Technologies) supplemented with 100 I.U./ml penicillin, 100 ug/ml streptomycin (P/S), 10 units/ml heparin and 5% fetal calf serum (FCS) (all supplements also obtained from Life Technologies). Spleen and kidney were collected and single cell suspensions generated using 100 μm nylon cell strainers (BD Biosciences). Blood cell suspensions were placed onto 51% Percoll (GE Healthcare) cushions whereas kidney and spleen suspensions were placed onto 30/51% discontinuous density gradients. All suspensions were then centrifuged at 500 × *g* for 30 min at 4 °C. The interface cells were collected and washed twice with L-15 containing 5% FCS.

### Production of rainbow trout recombinant IL-6

The sequence encoding the mature peptide of trout IL-6^69^ was amplified from spleen cDNA prepared from *Aeromonas salmonicida* infected fish^70^ and cloned to the pET/Duet-1 vector (Novagen, UK). The N-terminal ATG (M) codon and a C-terminal His tag (GSGHHHHHHHHHH) were incorporated from the vector for translation initiation and for purification of the recombinant protein. Thus, the rtIL-6 had 211 amino acids, with a predicted molecular weight of 23.8 kDa and pI of 7.27. Sequence analysis of three clones (pET/IL-6a, b, c) revealed that they were identical but differed by four nucleotides, of which two resulted in two amino acid changes from the published IL-6^69^, presumably due to polymorphism. The sequence has been submitted to EMBL/GenBank/DDBJ databases under the accession number FR715329. A plasmid was used to transform BL21 Star (DE3) competent cells (Invitrogen) and protein expression was induced by IPTG and purified under denaturing conditions as described previously^71^. The purified, denatured rtIL-6 was refolded in refolding buffer containing 50 mM Tris–HCl, pH8.0, 0.5 M arginine, 0.5% Triton-100 and 5 mM β-mercaptoethanol at 4 °C for 2 days. The refolded rtIL-6 was repurified under native conditions and eluted at concentrations of up to 0.5 mg/ml. LPS contamination was checked by examining the expression of a number of LPS-responsive genes, including IL-1β, TNF-α, IL-8, IL-10 and IL-11, and by undertaking the *Limulus amoebocyte* lysate assay (Sigma) as per the manufacturer’s instructions.

### Cell stimulation

Total leukocyte populations were dispensed in 24-well plates at a density of 2 × 10^6^ cells per ml and incubated with the appropriate stimulus: recombinant trout IL-6 (200 ng/ml), LPS (100 μg/ml; >95% purity) (Sigma), TNP-KLH (5 μg/ml) (Biotools), TNP-LPS (5 μg/ml) (Biotools) or anti-IgM Ab (2 μg/μl) (clone 1.14)^72^ at concentrations previously optimized. Non-stimulated controls were always included. Cells were always incubated at 20 °C for different periods of time depending on specific experiments.

### Cell sorting

IgM^+^ B cells were sorted from spleen, blood or kidney leukocyte suspensions using a BD FACSAria III (BD Biosciences) cell sorter. For this, leukocytes were incubated for 30 min on ice with an anti-trout IgM mAb (1.14) coupled to phycoerythrin (PE) in Staining buffer (PBS containing 1% FCS and 0.5% sodium azide) that prevents cell activation. Following two washing steps, cells were resuspended in FACS buffer and IgM^+^ B cells isolated based on their FSC/SSC profiles (to exclude the granulocyte gate) and then on the basis of the fluorescence emitted by the anti-trout IgM antibody. IgM^+^ and IgM^−^ cells were then collected in Trizol for subsequent RNA isolation.

### Real time PCR analysis of sorted cells

Total RNA was isolated from IgM^+^ sorted populations using Tri-reagent (Life Technologies). The cDNA synthesis and real-time PCR analysis were performed as described previously^33^,^71^. The primers (Supplementary Table S1) for real-time-PCR were designed so that at least one primer crossed an intron, to ensure that genomic DNA could not be amplified under the PCR conditions used. The expression level of each gene was first normalized against the expression level of EF-1α and then expressed as a fold change that was calculated as the average expression of the IL-6/LPS stimulated samples divided by that of the controls.

### Detection of phosphorylated STAT3

To confirm the phosphorylation of STAT3 in IL-6 and LPS-treated B cells and demonstrate direct effects on IgM^+^ B cells, we first sorted IgM^+^ cells from splenocyte cultures as described above and then incubated them with the different stimuli in the absence of other cell types. For this, sorted cells adjusted to 2 × 10^6^ cells per ml were disposed in 96 well plates and incubated with IL-6 (200 ng/ml), LPS (100 μg/ml) or media alone for 1 h at 20 °C. After the incubation, cells were lysed using RIPA buffer containing protease inhibitors (Roche). Proteins were fractionated onto a denaturing 12% SDS-PAGE gel and transferred onto a polyvinylidene difluoride (PVDF) membrane (Immobilon-P; Millipore Merck). After blocking in PBS with 5% skim milk for 1 h, the membrane was incubated with a mAb against phospho-STAT3 (Ser727) (Santa Cruz Biotechnology) in blocking solution at 4 °C overnight. After three washing steps, the membrane was incubated for 1 h with the secondary antibody, a goat anti-mouse IgG-HRP conjugate (GE Healthcare Life Sciences). The reactive bands were visualized with the ECL system (GE Healthcare Life Sciences).

### B cell proliferation

The BrdU Flow Kit (Becton Dickinson) was used to measure the proliferation of IgM^+^ cells following manufacturer’s instructions. Splenocytes at a concentration of 2 × 10^6^ cells per ml were incubated for 3 days at 20 °C with the different stimuli as described above. Bromodeoxyuridine (BrdU, 10 μM) was then added to the cultures and the cells were incubated for an additional 24 h. After that time, trout cells were collected and stained with anti-IgM-PE (1.14) antibody and then fixed and permeabilized with Cytofix/Cytoperm Buffer for 15 min on ice. Afterwards, cells were incubated with Cytoperm Permeabilization Buffer Plus for 10 min on ice and re-fixed with Cytofix/Cytoperm Buffer for 5 min at RT. Cells were then incubated with DNase (30 μg/10^6^ cells) for 1 h at 37 °C to expose the incorporated BrdU. Finally, cells were stained with FITC anti-BrdU antibody for 20 min at RT and analysed by flow cytometry (BD FACSCalibur, BD Biosciences).

### ELISPOT analysis

ELISPOT was used to quantify the number of IgM-secreting B cells. For this, ELISPOT plates containing Inmobilon-P membranes (Millipore) were activated with 70% ethanol for 30 s, coated with anti-trout IgM mAb (clone 4C10) at 2 μg/ml in PBS and incubated overnight at 4 °C. To block non-specific binding to the membrane, plates were then incubated with 2% bovine serum albumin (BSA) in PBS for 2 h at RT. Stimulated (with IL-6 or LPS as a positive control) or unstimulated splenocytes from individual fish were added to the wells in triplicate at a concentration of 1 × 10^5^ cells per well. After 72 h of incubation at 20 °C, cells were washed away 5 times with PBS and plates were blocked again with 2% BSA in PBS for 1 h at room temperature. After blocking, biotinylated anti-trout IgM mAb (clone 4C10) was added to the plates and incubated at 1 μg/ml for 1 h at RT. Following additional washing steps (5 times in PBS) the plates were developed using streptavidin-HRP (Thermo Scientific) at RT for 1 h, washed again with PBS and incubated with 3-amino 9-ethylcarbazole (Sigma Aldrich) for 30 min at RT in the dark. Substrate reaction was stopped by washing the plates with tap water. Once the membranes had dried, they were digitally scanned and spot counts determined by the ImmunoSpot Series 45 Micro ELISPOT Analyzer.

### Determination of total IgM levels

To determine total (both intracellular and extracellular) IgM levels, cells were fixed for 5 min with 4% paraformaldehyde in PBS, then permeabilized for 30 min in permeabilizitation buffer (staining buffer containing 0.1% saponin) and thereafter incubated with an anti-IgM-PE antibody in permeabilization buffer for another 30 min. After incubation, cells were washed three times with staining buffer and analyzed by flow cytometry.

### NF-kB activation

Trout splenocytes were isolated and seeded in complete MGFL-15 medium (MGFL-15 supplemented with P/S, 100 μg/ml gentamicin, 10% newborn calf serum (Gibco) and 5% carp serum). Cells were incubated for 24 h with complete media containing IL-6, LPS or control media alone. Following stimulation, cells were fixed in 1% formaldehyde, and washed twice in PBS with 2% calf serum and 0.1% saponin (permeabilization buffer). To determine nuclear translocation in IgM^+^ cells, splenocytes were stained with anti-p65 (Santa Cruz Biotechnology) and anti-trout IgM (1.14) for 30 min at 4 °C followed by 20 min at RT. Following the primary staining, cells were washed and stained with goat anti-rabbit APC (Jackson ImmunoResearch) and rabbit anti-mouse FITC (Jackson ImmunoResearch). Prior to acquisition, Hoechst33342 nuclear stain (Molecular Probes) was added as per the manufacturer’s recommendations. Data were collected on an ImageStream MKII and analyzed using IDEAS software (Amnis), as described previously^11^.

### Analysis of MHC-II expression

The levels of MHC-II expression on the surface of IgM^+^ B cells were measured via flow cytometry using a mAb against trout MHC-II^73^. Stimulated or unstimulated splenocytes were washed in Staining Buffer and co-incubated with PE-conjugated anti-trout IgM and the Alexa 647-conjugated anti-MHC-II antibody for 30 min at 4 °C protected from light. Finally cells were washed twice with the same buffer and analysed by flow cytometry.

### Phagocytic activity

Purified splenocytes (2 × 10^6^) were cultured in 1 ml/well in 24-well plates in the presence or absence of IL-6 or LPS for 24, 48 or 72 h. After each time point, cells were incubated for 16 h at 20 °C with fluorescent beads (FluoSpheres Microspheres, 1.0 μm, Crimson Red Fluorescent 625/645, 2% solids; Life Technologies) at a cell/bead ratio of 1:10 or without beads as negative controls. Non-ingested beads were removed by centrifugation (100 × g for 10 min at 4 °C) over a cushion of 3% (w/v) BSA (Fraction V; Fisher Scientific) in PBS supplemented with 4.5 (w/v) D-glucose (Sigma). Afterwards, cells were resuspended in staining buffer, labeled with PE-anti-IgM mAb (30 min at 4 °C), washed with the same buffer and analyzed by flow cytometry.

### Calcium flux

For calcium flux analysis, the calcium indicator Fluo-3 AM (Life Technologies) was used, following the manufacturer’s instructions. Briefly, Fluo-3 was dissolved in DMSO and further diluted in an equal volume of 20% (w/v) Pluronic F-127 (Life Technologies). Splenocytes were cultured in the presence or absence of LPS or IL-6 during 24, 48 or 72 h. After each time point, cells were diluted in L-15 medium without FCS and incubated with Fluo-3 AM at a final concentration of 5 μM for 1 h. Cells were then collected and washed, and a baseline reading for 30 s acquired in a FACSCalibur flow cytometer. Then 0.5 μg/ml of anti-IgM were added to the tube, and the emission of fluorescence (525 nm) determined for 180 s in each sample.

### Bioactivity of IL-6 *in vivo*

To assess the bioactivity of rtIL-6 *in vivo*, rainbow trout of approximately 5–7 g were divided into three groups of 24 fish and injected intraperitoneally (i.p.) with 100 μl of PBS, 100 μl of PBS with IL-6 (100 ng/fish), 100 μl of PBS containing 2 × 10^10^ TCID_50_/ml inactivated IPNV (kindly donated by Professor Øystein Evensen), or 100 μl of PBS with a combination of IPNV and IL-6 at the same concentration as above. At days 2, 6 and 15, six trout from each group were killed by benzocaine overdose. Blood was collected from the caudal vein to determine antibody concentration and thereafter splenocytes and kidney leukocytes were isolated to determine the number of IgM-secreting cells in ELISPOT assays as described above.

### Antibody production

Serum samples were obtained after blood clotting at RT for 1–2 h followed by incubation overnight at 4 °C. Afterwards, the clot was centrifuged at 4000 rpm for 10 min and serum samples were collected in a new tube that was centrifuged again at 10000 rpm for 10 min. Supernatants were finally collected in different tubes and stored at −20 °C until use. The production of total IgM, IPNV-specific IgM and natural IgM antibodies (with reactivity to TNP-BSA, galactosidase, phosphorylcholine and poly I:C) was then established by capture ELISA.

To assess total IgM levels, 96-well ELISA plates were coated overnight with 100 μl of 2 μg/ml mouse anti-trout Ig mAb 4C10. Wells were then blocked with 100 ul of 1% BSA in 1% Tween-20 PBS for 1 h at RT. Plates were washed 3 times with PBS-1% Tween-20 and serum samples were diluted 1:100 in PBS-1% BSA and added to the wells. Samples were incubated 1 h at RT and washed 3 times in PBS-1% Tween-20. Then, 50 μl of biotinylated 4C10 mAb (1 μg/ml) diluted in blocking buffer were added to the wells and samples incubated for 1 h at RT. After three washing steps, plates were incubated with 50 μl of Streptavidin-HRP (1:2000 in PBS-1% BSA) for 1 h at RT. Wells were washed again 3 times and then 50 μl of TMB substrate (Sigma) were added. Absorbance at OD_405_ was measured in a FLUO Star Omega Microplate Reader.

An ELISA method was also used to measure IPNV-specific antibodies. Plates were coated with 100 μl of polyclonal anti-IPNV antibody diluted at 1:5000 in diluent buffer (1% fat free dry milk-PBS) and incubated overnight at 4 °C. Plates were washed 3 times in PBS-0.05% Tween-20 and blocked with 5% fat free dry milk-PBS for 2 h at RT. After 3 washing steps, serum samples were diluted at 1:100 in diluent buffer and incubated overnight at 4 °C. After that, biotinylated 4C10 mAb and Streptavidin-HRP were used as described before. OPD substrate was added to the wells and samples were measured at OD_492_ nm.

In order to measure the levels of natural IgM antibodies, plates were coated with 100 μl of 15 μg/ml of TNP-BSA, 10 μg/ml galactosidase, 20 μg/ml phosphorylcholine or 20 μg/ml poly I:C diluted in PBS and incubated overnight at 4 °C. Plates were then blocked with 100 μl of 1% BSA in 1% Tween-20 in PBS for 1 h at RT. After washing steps in PBS-1% Tween-20, the serum samples were diluted 1:100 in PBS-1% BSA and added to the wells. Biotinylated 4C10 mAb and Streptavidin-HRP were also used as described in total anti-IgM detection ELISA. Finally, OPD substrate was added to the wells and samples were measured at OD_492_ nm.

### Statistical analysis

Statistical analyses were performed using a two-tailed Student’s *t* test with Welch’s correction when the F test indicated that the variances of both groups differed significantly. The differences between the mean values were considered significant on different degrees, where * means *P* ≤ 0.05, ** means P ≤ 0.01 and *** means P ≤ 0.005 (GraphPad Prism 4 software).

## Additional Information

**How to cite this article**: Abós, B. *et al*. Distinct Differentiation Programs Triggered by IL-6 and LPS in Teleost IgM^+^ B Cells in The Absence of Germinal Centers. *Sci. Rep*. **6**, 30004; doi: 10.1038/srep30004 (2016).

## Supplementary Material

Supplementary Information

## Figures and Tables

**Table 1:** Summary table comparing the effects of IL-6 and LPS on trout spleen IgM^+^ B cells.

Immune function	IL-6	LPS
STAT3 transcription	Up-regulated	Up-regulated
SOCS3 transcription	Up-regulated	No effect
STAT3 phosphorylation	Activated	Activated
Mitogenic effects	Significant	Very significant
IgM secretion	Increased	Increased
mIgM expression	Increased	Increased
Total IgM expression	Increased	Increased
Blimp1 transcription	Up-regulated	No effect
ACKR2 transcription	Up-regulated	No effect
p65 translocation to nucleus	Yes	Yes
IL-1β transcription	No effect	Up-regulated
IL-8 transcription	No effect	Up-regulated
TNF-α transcription	No effect	Up-regulated
CATH1 transcription	No effect	Up-regulated
CATH2 transcription	Up-regulated	Up-regulated
Hepcidin transcription	No effect	No effect
BCR signaling to anti-IgM	Increased	Increased
MHC-II expression	Decreased	Increased
Phagocytic activity	No effect	Increased
CD80/86 transcription	No effect	Up-regulated
CD83 transcription	No effect	Up-regulated
