# Supplementary material for: Distinct Differentiation Programs Triggered by IL-6 and LPS in Teleost IgM+ B Cells in The Absence of Germinal Centers
Source: Sci Rep. 2016 Aug 2;6:30004. doi: 10.1038/srep30004 (PMC4969607; doi:10.1038/srep30004)

Supplemental information

DISTINCT DIFFERENTIATION PROGRAMS TRIGGERED BY IL-6 AND LPS IN TELEOST IgM^{+} B CELLS IN THE ABSENCE OF GERMINAL CENTERS

Beatriz Abós[1], Tiehui Wang[2], Rosario Castro[1], Aitor G. Granja[1], Esther Leal[1], Jeffrey Havixbeck[3], Alfonso Luque[1], Daniel Barreda[3], Chris J. Secombes[2], Carolina Tafalla[1*].

[1]*Centro de Investigación en Sanidad Animal (CISA-INIA), Madrid, Spain.*

[2]*Scottish Fish Immunology Research Centre, University of Aberdeen, Aberdeen, UK*

[3]*Department of Biological Sciences, University of Alberta, Alberta, Canada*

* Corresponding author: Carolina Tafalla. Email: tafalla@inia.es. Telephone: +34 916202300. Fax: +34 916202247.

Fig. S1. IgM+ cells constitutively express the IL-6 receptor subunits and are activated by IL-6. (**a**) Transcription levels of the IL-6 receptor α chain (IL-6Rα) and glycoprotein (gp)130 were evaluated in sorted IgM+ B cells from spleen, peripheral blood (PBLs) and kidney of non-stimulated fish. RNA was extracted from sorted IgM+ B cells and levels of transcription evaluated by real-time PCR in duplicate. Data from 3 independent fish are shown as mean gene expression level relative to the expression level of the endogenous control (EF-1α) + standard deviations. (**b**) Spleen leukocytes were incubated with media containing IL-6 (200 ng/ml), LPS (100 μg/ml) or control media alone for 24 h at 20ºC. After that time, IgM+ cells were sorted by using an anti-trout IgM mAb and RNA extracted. Relative transcript expression (mean + standard deviation, n=6) of STAT3 and SOCS3 is shown. Asterisks denote significant differences between cells treated with IL-6 or LPS and their corresponding controls. * $P < 0.05$, ** $P < 0.01$, ***, $P < 0.001$. (**c**) Western blot showing phosphorylation of STAT3 in sorted IgM+ cells incubated for 1 h with IL-6, LPS or media alone.

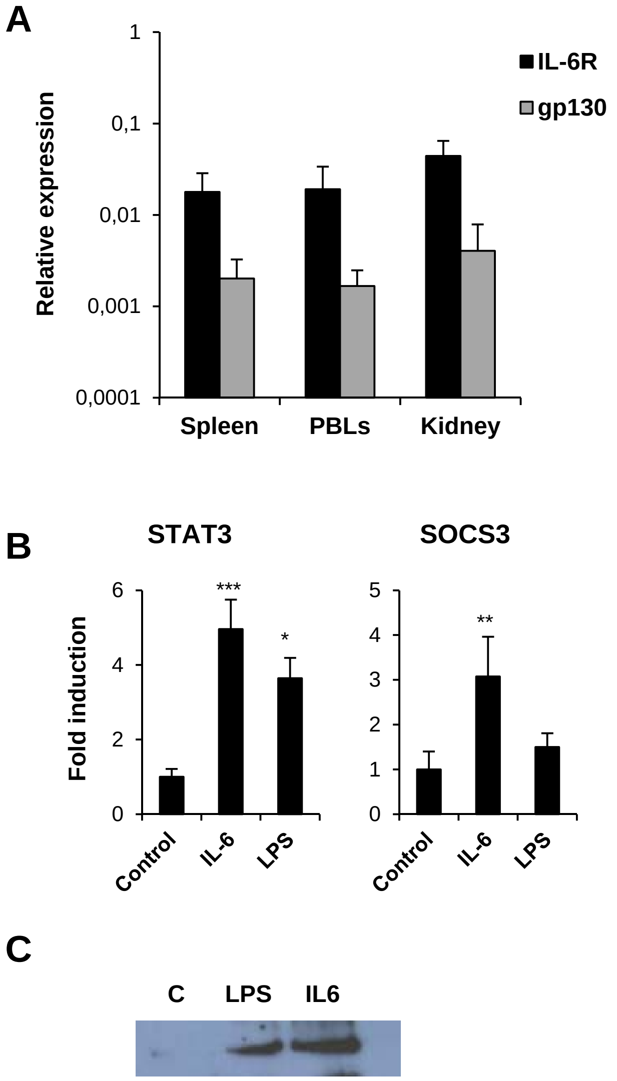

Table S1. List of primers used in this study to evaluate gene transcription.

Gene	Forward primer	Reverse primer	Acc. no.
EF-1α	CAAGGATATCCGTCGTGGCA	ACAGCGAAACGACCAAGAGG	AF498320
IL-6Rα	CAACACCTGGACAGCCCCTG	CACAGGAAGCAACCACCCACA	FN824530
gp130	GCGTCCTGTTTGTCATAGTGCTAACTG	CCTTGGGACTGACAGCTTTGGT	FN824531
STAT3	ACCAACCACCCCAAGAATGTGA	CCTTTACCCACCATGTTCTCTTTGC	OMU60333
SOCS3	CACAGAGAAACCGTTAAAAGGACTATCC	AAGGGGCTGCTGCTCATGAC	AM748723
Blimp1	GGCAGTGGACCTGTGGAAGG	CGCAGGTGGACCTTGAGGTT	CCAF010048522
ACKR2	TCAGAAACACTTTTTTTCCAGAGATATAC	GAGGGTGCAAATGATAATGTAAGAGAC	KM516350)
IL-1β1	CCTGGAGCATCATGGCGTG	GCTGGAGAGTGCTGTGGAAGAACATATAG	AJ278242
IL-8	TCCTGACCATTACTGAGGGGATGA	AGCGCTGACATCCAGACAAATCTC	AJ279069
TNF-α3	GCTGCACTCTTCTTTACCAAGAAACAAG	CCACTGAGGACTTGTAATCACCATAGGT	HE798544
CATH1	ACCAGCTCCAAGTCAAGACTTTGAA	TGTCCGAATCTTCTGCTGCAA	AY594646
CATH2	ACATGGAGGCAGAAGTTCAGAAGA	GAGCCAAACCCAGGACGAGA	AY542963
Hepcidin	GCTGTTCCTTTCTCCGAGGTGC	GTGACAGCAGTTGCAGCACCA	CA369786
CD80/86	CAGGAACACACTGTCTGCAGGC	CTGCTCCCTTTCCTCCTTGATTACTTC	EU927451
CD83	GTGAGGTGGTACAAGCTGGGTG	GCTGCCAGGAGACACTTGTACCT	AY263797

Fig. S2. Effect of IL-6 on IgM secretion in splenocytes from vaccinated fish. Rainbow trout were i.p. injected with 100 µl of PBS or 100 µl of PBS with 2 x 10^{10} $TCID_{50}$/ml inactivated IPNV. One week after injection, fish were killed and splenocytes obtained to compare the effect of IL-6 on IgM secretion on vaccinated and mock-vaccinated fish through ELISPOT. For this, splenocytes from vaccinated and mock-vaccinated fish were cultured for 3 days in ELISPOT plates previously coated with anti-trout IgM mAb (2 µg/ml) in the presence or absence of IL-6 (200 ng/ml). After incubation, cells were washed away and a biotinylated anti-trout IgM mAb (1 µg/ml) was used to detect numbers of spot forming cells. Quantification of spot forming cells are shown (mean + standard deviation; n= 7). Asterisks indicate significant differences between groups as indicated. * $P < 0.05$; ** $P < 0.01$; *** $P < 0.001$.

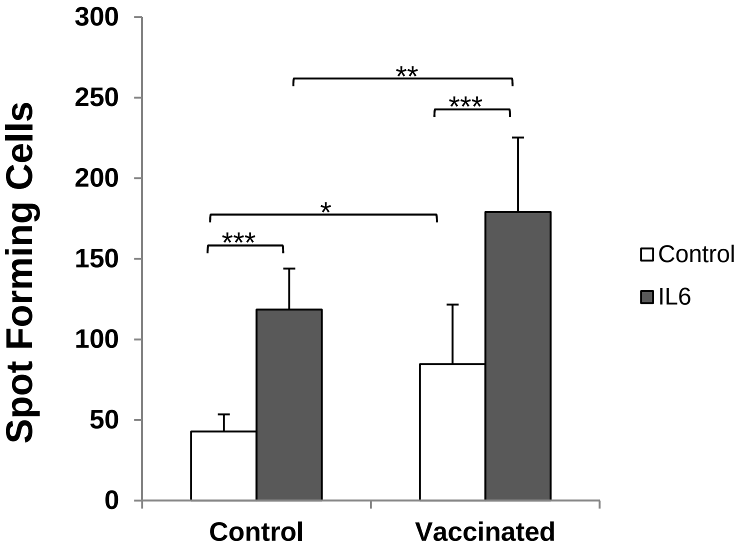

Supplement: Supplementary Information [file srep30004-s1.pdf]
